# Genes Associated With Prognosis After Surgery For Malignant Pleural Mesothelioma Promote Tumor Cell Survival *In Vitro*

**DOI:** 10.1186/1471-2407-11-169

**Published:** 2011-05-13

**Authors:** Gavin J Gordon, Raphael Bueno, David J Sugarbaker

**Affiliations:** 1 Division of Thoracic Surgery, Brigham and Women's Hospital, Harvard Medical School, 75 Francis St., Boston, MA 02115 USA

**Keywords:** mesothelioma, Cell biology/culture, Genes/polymorphisms, Genetics/genomics

## Abstract

**Background:**

Mesothelioma is an aggressive neoplasm with few effective treatments, one being cytoreductive surgery. We previously described a test, based on differential expression levels of four genes, to predict clinical outcome in prospectively consented mesothelioma patients after surgery. In this study, we determined whether any of these four genes could be linked to a cancer relevant phenotype.

**Methods:**

We conducted a high-throughput RNA inhibition screen to knockdown gene expression levels of the four genes comprising the test (ARHGDIA, COBLL1, PKM2, TM4SF1) in both a human lung-derived normal and a tumor cell line using three different small inhibitory RNA molecules per gene. Successful knockdown was confirmed using quantitative RT-PCR. Detection of statistically significant changes in apoptosis and mitosis was performed using immunological assays and quantified using video-assisted microscopy at a single time-point. Changes in nuclear shape, size, and numbers were used to provide additional support of initial findings. Each experiment was conducted in triplicate. Specificity was assured by requiring that at least 2 different siRNAs produced the observed change in each cell line/time-point/gene/assay combination.

**Results:**

Knockdown of ARHGDIA, COBLL1, and TM4SF1 resulted in 2- to 4-fold increased levels of apoptosis in normal cells (ARHGDIA only) and tumor cells (all three genes). No statistically significant changes were observed in apoptosis after knockdown of PKM2 or for mitosis after knockdown of any gene.

**Conclusions:**

We provide evidence that ARHGDIA, COBLL1, and TM4SF1 are negative regulators of apoptosis in cultured tumor cells. These genes, and their related intracellular signaling pathways, may represent potential therapeutic targets in mesothelioma.

## Background

Malignant pleural mesothelioma (MPM) is a highly lethal pleural cancer for which there are few effective treatments and overall >90% five-year mortality. Approximately 3,000 patients are diagnosed with MPM in the US annually and the incidence worldwide is projected to rise substantially[1-3] owing to a lack of regulatory oversight governing the use of asbestos, the most common cause for MPM (70-80%) [4-8].

MPM is characterized by multi-focal tumor growth originating in the parietal pleura and often accompanied by a pleural effusion. The tumor eventually spreads to involve the visceral pleura producing a rind that constricts the lung, heart and mediastinum. Presumably due to the tumor's location and rapid growth, death usually results from compression of vital mediastinal structures rather than from metastatic spread[7,9,10]. Without any treatment, the expected median survival of patients presenting with MPM is between 4 and 12 months. Unfortunately, survival has not improved during the past decade as MPM is exceedingly resistant to most chemotherapy regimens. Pemetrexed and cisplatin combination chemotherapy was shown in one of the few positive prospective randomized trials to be the best chemotherapy regimen for MPM. However, this therapy only increased median survival from 9 to 11 months[11]. Radiation therapy is generally ineffective as a primary treatment as well [10,12].

Surgery-based multi-modal therapy has been reported to improve survival in a subset of patients who present with early MPM which is confined to a single thoracic cavity. This treatment strategy includes a complete surgical resection of the tumor utilizing an extrapleural pneumonectomy followed by chemotherapy with paclitaxel (or pemetrexed) and cisplatin followed by whole chest external beam radiation therapy to eradicate any residual disease. Up to 40% of selected patients will survive for 5 years following treatment. The majority, but not all, of these long-term survivors have early stage disease as manifested by negative lymph node status, low tumor volume and have epithelial histology [10,13-19].

We have previously described[20] a molecular prognostic test that is based on select ratios of mRNA expression levels of 4 genes (TM4SF1, PKM2, COBLL1, ARHGDIA) to predict outcome after surgery for patients with MPM. Two of these genes (TM4SF1, COBLL1) are expressed at higher levels in tumors associated with a relatively good prognosis while the other two (ARHGDIA, PKM2) are expressed at relatively higher levels in tumors associated with a relatively poor prognosis. This test was subsequently found to differentiate between patients on the basis of postsurgical outcome in multiple independent retrospective cohorts[20,21]. Most recently, this test has been shown to independently predict overall survival and cancer-specific survival in a prospective clinical trial of patients undergoing surgery (i.e., extra-pleural pneumonectomy) for MPM[22]. These studies indicate that the genes comprising the test are effective molecular markers for two different MPM patient subsets, and thus could conceivably be used to rationally design clinical trials to test the efficacy of proposed treatments targeting these specific genes and related pathways. In this study, we have begun to explore this possibility by determining whether any of these 4 genes could be linked a cancer relevant phenotype (e.g., mitosis, apoptosis) using a high-throughput, cell-based RNA inhibition (RNAi) approach.

## Methods

### Cell lines and cell culture

The normal cell line WI38 and the tumor cell line A549 were purchased from the American Type Culture Collection (http://www.atcc.org). Both cell lines were grown in 384-well plates (4titude, Surrey, United Kingdom) under RPMI1640 medium (Invitrogen, Carlsbad, California) supplemented with 10% calf serum and antibiotics. A549 cells were grown in wells coated with collagen I (BD Biosciences, San Jose, California).

### Gene knockdown studies

A549 and WI38 cells were seeded at a density of 1,000 cells/well and 3,000 cells/well, respectively. One day post-seeding, cells were separately transfected with siRNAs (Applied Biosystems/Ambion *Silencer*^® ^siRNA library, Austin, Texas) targeting positive control genes (KIF11, PLK1) or experimental genes (ARHGDIA, COBLL1, PKM2, TM4SF1) using Lipofectamine RNAiMax (Invitrogen) at 0.09 μl/well. A single siRNA molecule was used for each positive control gene: KIF11 (siRNA ID #105925), PLK1 (siRNA ID #213548). Positive control genes were chosen to monitor the performance of the assay as each gene is widely known to affect both mitosis and apoptosis. Three structurally distinct siRNA molecules were separately used for each experimental gene to confirm specificity of effect: ARHGDIA (siRNA IDs #119694, #119695, #119907), COBLL1 (siRNA IDs #136636, #136637, #136638), PKM2 (siRNA IDs #285, #286, #287), TM4SF1 (siRNA IDs #144307, #144308, #144309). All siRNAs were used at a final concentration of 30 nM. Negative controls consisted of scrambled, non-specific siRNAs and mock transfection (i.e., transfection mix only). Positive and negative controls were placed at multiple positions on the plate to control for positional effects (if any). Each cell line/siRNA molecule combination or cell line/mock transfection combination was conducted in triplicate, i.e., 3 wells per plate. All experiments were run in triplicate with a single experiment consisting of one plate to be used to assess knockdown efficiency (via quantitative RT-PCR) and a separate identical plate to be used for phenotypic and morphological assays described below.

### Phenotypic studies of gene knockdown cell lines

Assessment of apoptosis and mitosis was conducted using a high-throughput approach [23]based on computer-assisted automated analysis of captured microscopic digital images [24,25], conducted as part of a fee-for-service arrangement with Cenix Bioscience (http://www.cenix-bioscience.com/). General strategies to minimize false discoveries were used as previously described [26]. Essentially, siRNA-transfected and control adherent cells were fixed at 72-hours post-transfection using 4% paraformaldehyde in PBS, washed using PBS, and stored at 4°C until analyzed further. Detection of apoptotic and mitotic molecular markers was conducted using immunological assays after blocking in a buffer containing 0.1% Triton, 0.01% Saponin, and 4% BSA in PBS. Primary antibody solutions (1:200 rabbit anti-cleaved lamin A in blocking buffer for apoptosis; 1:900 mouse anti-phospho-histone H3 in blocking buffer; 1:100 rabbit anti-alpha-tubulin in blocking buffer to show cellular morphology) were applied overnight at 4°C. All primary antibodies were obtained from Cell Signaling Technology (Danvers, Massachusetts). After washing twice in PBS, a secondary antibody solution for was applied for 2 h at RT (goat anti-rabbit alexa488, goat anti-mouse alexa555, both antibodies from Invitrogen in a dilution of 1:1,000 + 1 μg/ml Hoechst 33342, in blocking buffer). Cells were washed three times in PBS and images were captured using an ImageXPress Micro automated microscope, with 10x objective, binning 2, 4 sites per well, Dapi/FITC/Cy3/Cy5 channels. QC analysis of digitally captured visual images containing fluorescent signals corresponding to apoptotic and mitotic molecular markers, as well as Hoechst staining of cellular DNA, was conducted using MetaMorph software (MDS Analytical Technologies, Downingtown, Pennsylvania). Quantitative auto-image analysis was conducted using eCognition software (Definiens, Parsippany, New Jersey) with rule sets optimized using data from positive control experiments.

### Data quantification and analysis

Raw image data generated above was imported into Spotfire DXP software (Tibco, Palo Alto, California) to remove from consideration areas with high background, bad focus, and/or image haze. Images were quantified by first calculating a individual well average from 4 sites/well then calculating a triplicate average from the three well averages, per sample, per readout. Experimental data was then normalized (to 100%) using the average of negative control data per sample, per readout. For statistical analysis, the following indices were calculated: Apoptotic Index ({[number of cleaved lamin A positive cells]/[number of nuclei]}*100%), Mitotic Index ({[number of phospho-histone H3 positive cells]/[number of nuclei]}*100%), Condensed Chromatin Index ({[number of condensed chromatin positive cells]/[number of nuclei]}*100%), Aberrant Nuclei Index ({[number of aberrant nuclei]/[number of nuclei]}*100%), and the Cell Number Index (absolute number of nuclei). The Apoptotic and Mitotic Indices required co-localization of Hoechst stain and cleaved lamin A or phosphor-histone H3, respectively. The Condensed Chromatin and Aberrant Nuclei Indices were based on Hoechst stain intensity and nuclear shape parameters, respectively, that were defined based on analysis of positive control gene knockdown studies. Statistical analysis was performed using a two-sided Student's (parametric) t-test for pair-wise comparisons of average experimental and negative control index values. Data was initially collected and analyzed for only the Apoptotic and Mitotic Index. Therefore, to take into account multiple hypothesis testing, statistical significance at the *P *< 0.05 level was estimated by applying a corrective factor to experimental *P *values such that statistical significance was called when *P *< 0.00069 (i.e., 0.05/72 where 72 = 2 (cell lines) × 2 (index readouts) × 3 (siRNAs per gene) × 6 (genes). Uncorrected *P *values were used to evaluate subsequent indices (i.e., Condensed Chromatin, Aberrant Nuclei, Cell Number) since these data were analyzed only in tumor cells for the genes/siRNAs with statistically significant differences (i.e., *P *< 0.00069) previously identified in the Apoptotic Index.

### Quantitative RT-PCR

Total RNA was extracted from 384-well plates using lysis buffer and the Invisorb kit (both from Invitek, Berlin, Germany). RT reactions were set up using Applied Biosystems (Foster City, California) HighCapacity cDNA reagents and random hexamer primers following the manufacturer's recommended protocol. PCR reactions were run on an Applied Biosystems 7900HT device using a SYBR Green fluorometric-based detection system (SensiMix 2X PCR master mix, Quantace, Freising, Germany). The total reaction volume was 11 μl and contained 500 nM of target specific primers[22]. Gene expression levels in experimental knockdown cell lines were expressed as a percentage of parental control.

## Results

### Targeting of MPM prognostic genes using RNAi

**Figure 1:**
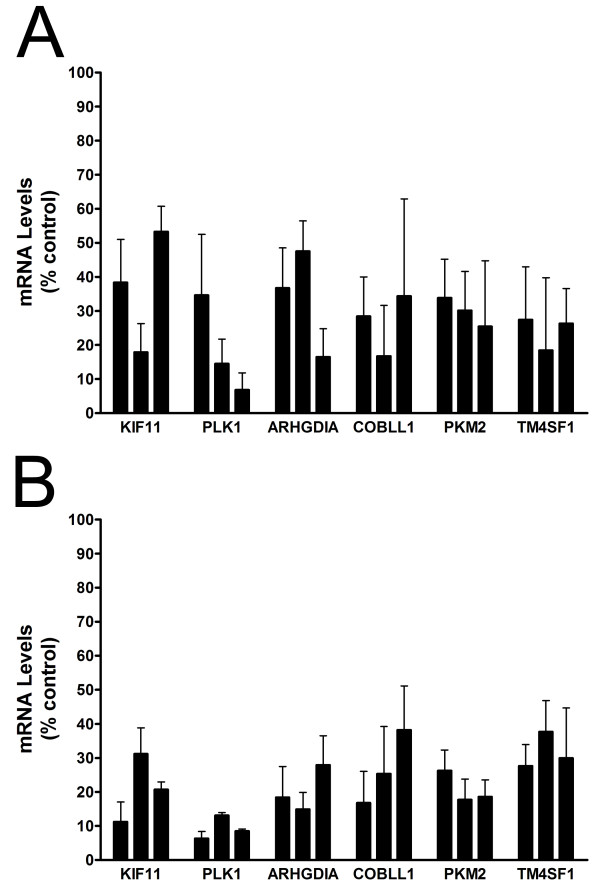
**RNAi-mediated knockdown of gene expression levels in cultured cells**. Levels of mRNA in normal (A) and tumor (B) cells after transfection with 3 different siRNA molecules per gene as represented by individual bars and expressed relative to untransfected (parental) control cells. Error bars, SEM.

All 6 genes (2 positive control and 4 experimental) were successfully targeted using 3 different siRNAs per gene as shown by reduced mRNA levels in normal and tumor knockdown cells relative to untransfected (parental) cells (Figure 1). Negative control cells transfected with non-specific (i.e., scrambled) siRNAs were not associated with changes in mRNA levels for any gene (data not shown). Gene expression levels for positive controls (KIF11, PLK1) and MPM prognostic genes (ARHGDIA, COBLL1, PKM2, TM4SF1) were reduced by ~50-80% in normal cells (Figure 1A). For reasons that were not immediately clear, a slightly more efficient knockdown was achieved in tumor cells (Figure 1B), as reflected by modestly reduced variability and lower overall mRNA levels.

### Detection of apoptosis and mitosis

**Figure 2:**
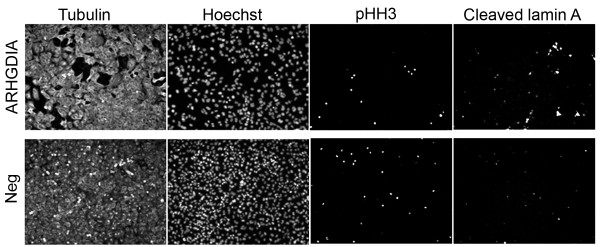
**Phenotypic analysis of ARHGDIA knockdown cell lines**. Tumor cells were transiently transfected with siRNAs targeting either ARHGDIA (top panels) or non-specific siRNAs (bottom panel, "Neg"). Cells were fixed and fluorescently labeled 72 hours later using primary antibodies to alpha-tubulin ("Tubulin") to show cellular morphology only, phospho-histone H3 ("pHH3") to mitosis, or cleaved lamin A to detect apoptosis. Nuclei were visualized using Hoechst stain. No obvious differences were observed in the numbers of pHH3-labeled cells in knockdown cells relative to negative control (based on total cell number) while the number of cleaved lamin A-positive cells were clearly increased in knockdown cells relative to negative control.

**Figure 3:**
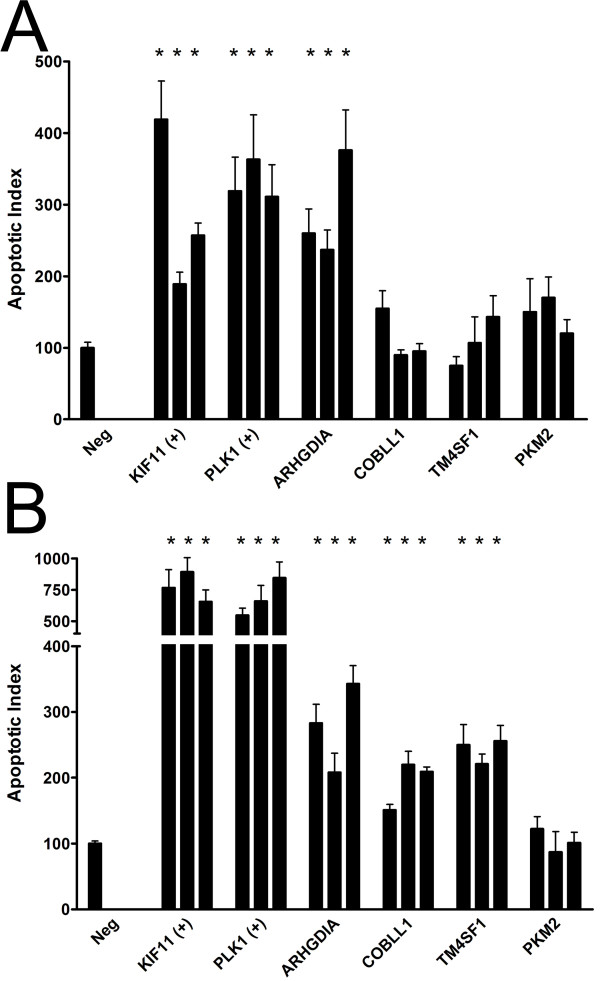
**Quantification of apoptosis after knockdown of MPM prognostic genes in cultured cells**. The Apoptotic Index was calculated as described in the Methods in normal (A) and tumor (B) cells transfected with 3 different siRNA molecules per gene (as represented by individual bars) targeting MPM prognostic genes ("ARHGDIA", "COBLL1", "TM4SF1", "PKM2"), positive control genes ("KIF11", "PLK1"), or non-specific siRNAs as negative controls ("Neg"). Error bars, SEM. *, statistically significant (*P *< 0.00069) relative to negative control.

Knockdown of ARHGDIA, COBLL1, and TM4SF1 resulted in increased levels of apoptosis in normal cells (ARHGDIA only) and tumor cells (ARHGDIA, COBLL1, TM4SF1) relative to negative controls. No changes were observed in apoptosis after knockdown of PKM2 or for mitosis after knockdown of any gene experimental gene. A representative image is shown in Figure 2 for ARHGDIA knockdown in tumor cells. To quantify these differences, we calculated Apoptotic and Mitotic Indices and found that apoptosis was statistically significantly elevated ~2- to 4-fold for silencing ARHGIA compared to negative control (siRNA1 *P *= 0.00067, siRNA2 *P *= 0.00098, siRNA3 *P *= 8.7 × 10^-10^) in normal cells (Figure 3A). Silencing of other experimental genes were in some cases associated with a modestly elevated degree of apoptosis that, as expected, did not achieve statistical significance for any siRNA (Figure 3A). A similar statistically significant 2- to 4-fold increase in the degree of apoptosis was observed after knockdown of ARHGDIA (siRNA1 *P *= 1.5 × 10^-6^, siRNA2 *P *= 1.2 × 10^-8^, siRNA3 *P *= 5.5 × 10^-9^), COBLL1 (siRNA1 *P *= 1.3 × 10^-5^, siRNA2 *P *= 1.8 × 10^-5^, siRNA3 *P *= 0.00063), and TM4SF1 (siRNA1 *P *= 7.3 × 10^-5^, siRNA2 *P *= 5.3 × 10^-5^, siRNA3 *P *= 3.2 × 10^-5^), but not PKM2, in tumor cells (Figure 3B). Despite a clear mitotic arrest after knockdown of positive control genes, as expected no statistically significant changes were observed in the Mitotic Index for any experimental gene relative to negative controls (data not shown).

### Morphological assessment of knockdown cell lines

**Figure 4:**
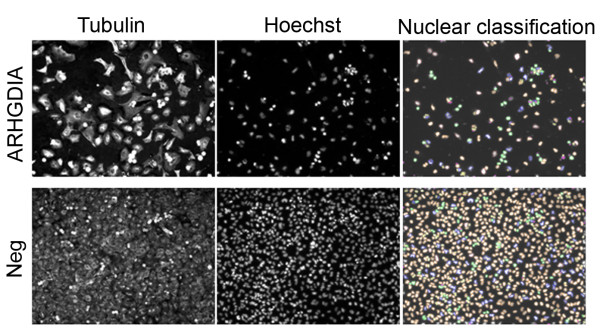
**Morphological appearance of ARHGDIA knockdown cell lines**. Tumor cells were transiently transfected with siRNAs targeting either ARHGDIA (top panels) or non-specific siRNAs (bottom panel, "Neg"). Cells were fixed and fluorescently labeled 72 hours later using primary antibodies to alpha-tubulin ("Tubulin") to show cellular morphology only. Nuclei were visualized using Hoechst stain and classified as either having condensed chromatin indicative of early apoptosis (green color, rightmost panels) and/or aberrantly shaped as a general indicator of cell well-being (blue color, rightmost panels). No obvious differences were seen in the relative numbers of aberrantly shaped nuclei relative to negative control while the numbers of cells containing condensed chromatin were increased in knockdown cells relative to negative control.

**Figure 5:**
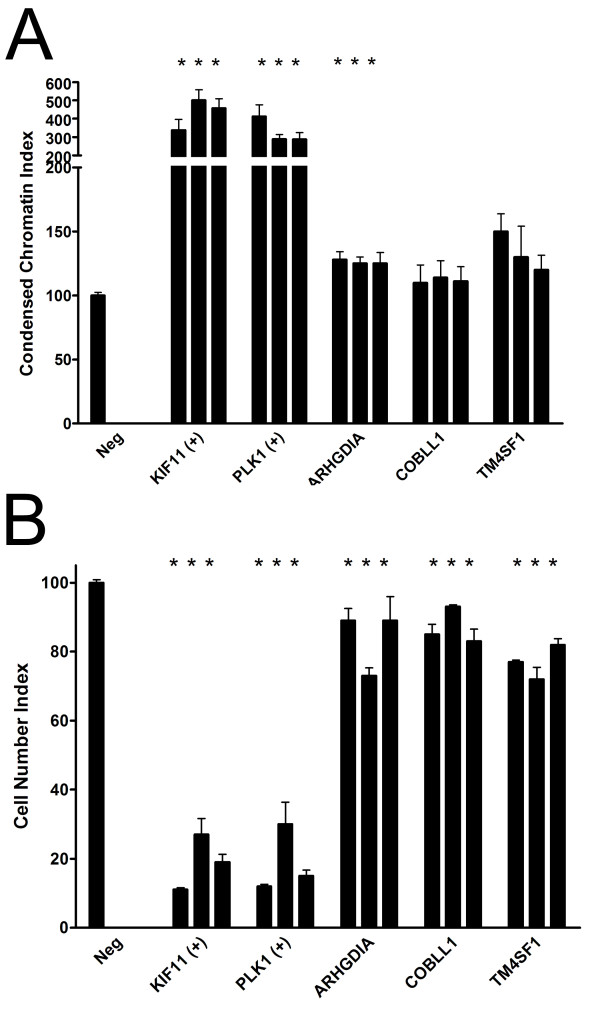
**Quantification of cultured cells displaying aberrant morphology after knockdown of MPM prognostic genes**. The Condensed Chromatin Index (A) and Cell Number Index (B) were calculated as described in the Methods in tumor cell transfected with 3 different siRNA molecules per gene (as represented by individual bars) targeting MPM prognostic genes ("ARHGDIA", "COBLL1", "TM4SF1"), positive control genes ("KIF11", "PLK1"), or non-specific siRNAs as negative controls ("Neg"). Error bars, SEM. *, statistically significant (*P *< 0.05) relative to negative control.

We examined nuclear morphology in experimental and control knockdown cell lines to look for relatively early indicators of apoptosis in order to complement studies described above in which apoptosis was measured using a relatively late indicator (e.g., cleaved lamin A). The numbers of cells with condensed nuclei, a hallmark of apoptosis, were increased in all positive control and knockdown cell lines that were previously associated with increased apoptosis as measured using cleaved lamin A, (e.g., ARHGDIA, COBLL1, TM4SF1) but not in the single knockdown cell line (PKM2) that was not. No statistically significant changes were observed in the numbers of aberrantly shaped nuclei under any circumstances by comparison of values calculated for the Aberrant Nuclei Index between experimental and controls (data not shown). A representative image is shown in Figure 4 for ARHGDIA knockdown in tumor cells. To quantify these differences, we calculated Condensed Chromatin and Aberrant Nuclei Indices. We found that ARHGDIA, COBLL1, and TM4SF1 knockdown cells were associated with a modest increase in the Condensed Chromatin Index (Figure 5A), although the results were statistically significant (*P *< 0.05) for siRNAs associated with a single experimental gene only (ARHGDIA; siRNA1 *P *= 0.004, siRNA2 *P *= 0.001, siRNA3 *P *= 0.001). As expected, no experimental gene/siRNA combination from the Aberrant Nuclei Index was statistically significantly (*P *< 0.05) different from the negative control.

### Quantification of cell numbers in knockdown cell lines

To generate a Cell Number Index, we used absolute cell numbers (via enumeration of Hoechst-stained nuclei) as a surrogate to determine the effects, if any, of experimental gene knockdown on cellular proliferation. We found that cell numbers decreased (relative to negative controls) in ARHGDIA, COBLL1, and TM4SF1 knockdown cells (Figure 5B) in support of previous results showing an increase in apoptosis under the same conditions. The observed decrease in cell number was statistically significant (*P *< 0.05) for all siRNAs for each experimental gene (gene, individual siRNA *P *value): ARHGDIA, siRNA1 *P *= 0.012, siRNA2 *P *= 2.0 × 10^-12^, siRNA3 *P *= 0.039; COBLL1, siRNA1 *P *= 0.008, siRNA2 *P *= 0.0006, siRNA3 *P *= 0.008; TM4SF1, siRNA1 *P *= 2.0 × 10^-19^, siRNA2 *P *= 2.0 × 10^-5^, siRNA3 *P *= 2.0 × 10^-11^.

## Discussion

Using an RNAi approach, we conducted *in vitro *gene knockdown studies of the 4 genes comprising an MPM prognostic test[20-22] and determined the effect, if any, on cell death and growth arrest using molecular markers for apoptosis and mitosis, respectively. Supporting data was obtained in select positive experiments using cellular morphology. We have found that three of the MPM prognostic genes (ARHGDIA, COBLL1, TM4SF1) are associated with a phenotype consistent with a negative regulator of apoptosis; rates of programmed cell death increased ~2- to 4-fold when mRNA expression levels for these genes were attenuated in cultured cells. Increased apoptosis was also associated with concurrent changes in nuclear morphology and a modest, but statistically significant, reduction in cell number. The fourth MPM prognostic gene (PKM2) could not be linked to either apoptosis or growth arrest in the current study.

We established the Hoechst-based Condensed Chromatin Index as an apoptotic indicator secondary to the detection of cleaved lamin A, a well established molecular marker[27]. We found that rates of apoptosis measured using this index were of a generally lower magnitude compared to those obtained via detection of cleaved lamin A, and furthermore not all genes linked to apoptosis via cleaved lamin A were associated with a statistically significant change in the Condensed Chromatin Index (e.g., COBLL1, TM4SF1), despite a trend in the expected direction. This is likely due to the facts that i) the index is based on clearly detectable nuclear changes that are a hallmark of relatively early events in apoptosis, such as local chromatin condensation while cleaved lamin-A is a relatively late event, and ii) we collected readouts at a single timepoint only (72 hours post-siRNA transfection) by which point early apoptotic events may have already occurred.

A second morphological readout (Aberrant Nuclei Index) was collected to determine whether experimental knockdown cells were associated with abnormally shaped nuclei. The Aberrant Nuclei Index detects cells whose nuclei deviate from the norm using two parameters, circularity and elliptical fit, which measure how spherical and how oval nuclei are respectively. Changes in nuclear morphology can be elicited by a number of different factors including, but not limited to fundamental alterations to microfilament arrangement and the nuclear skeleton. In addition to providing basic nuclear shape and stability, nuclear support proteins are also required for chromatin organization, transcription regulation, DNA replication, nuclear assembly, nuclear positioning, and apoptosis. Thus, the aberrant nuclei classification parameter may be best described as a general marker for cell well-being. We hypothesized that knockdown of MPM prognostic genes that led to an increase in apoptosis would also lead to an increase in the numbers of aberrantly shaped nuclei. Although the frequency of aberrantly shaped nuclei was modestly elevated under these expected conditions, the results were not statistically significant. The implication of this negative result, if any, is not clear and may relate to timing issues and/or other factors since only 1 of the 2 apoptosis positive control knockdown cell lines displayed a statistically significant increase in the numbers of aberrantly shaped nuclei.

The MPM prognostic genes examined in the current study have in some cases been previously linked to cancer or cancer relevant processes. TM4SF1, a.k.a. L-6 tumor antigen, is a distant member of the transmembrane 4 superfamily of cell-surface proteins that are characterized by the presence of four hydrophobic domains. Although the precise function and physiological role of this gene is unknown, the fact that it is a cell surface antigen that is highly expressed in different carcinomas and expressed at relatively low levels (if at all) in many normal tissues has led to its evaluation as a candidate therapeutic target for radioimmunotherapy treatment, particularly for breast cancer[28]. More recently, TM4SF1 has been linked to metastasis[29] and angiogenesis[30], but not apoptosis (to our knowledge) prior to the current study.

The COBLL1 (COBL-like 1) gene was cloned in 1999 and originally designated KIAA0977[31]. The deduced protein contains 1,166 amino acids and was found to be expressed at high levels in lung, liver, kidney, pancreas, ovary, spinal cord, brain, fetal liver, and all specific adult brain regions[31]. In 2003, Carroll et al. renamed the gene COBBL1 based on homology to the newly discovered COBL (i.e., cordon-bleu homolog, mouse) gene[32]. The specific role(s) of both genes are not known, but each is presumed to play a role in embryogenesis based on temporal expression patterns during development[32]. Given the known role of apoptosis in determining tissue morphology during normal embryonic development and the results of the current study, it is reasonable to hypothesize that inappropriate expression in tumor cells acts to prevent programmed cell death and promotes tumor cell survival.

The ARHGDIA (Rho GDP dissociation inhibitor [GDI] alpha) gene, first described in 1993[33], belongs a family of genes whose members regulate (Ras superfamily) Rho genes by keeping them in the inactive GDP-bound state[34]. ARHGDIA was the only gene in our RNAi studies found to impact apoptosis in both normal and tumor cells, with a generally greater magnitude of effect in tumor cells compared to the other 2 positive genes. Other investigations of ARHGDIA in the context of cancer have indicated a role in estrogen receptor signalling (and estrogen responsiveness) in breast cancer[35,36] and association with poor prognosis in colorectal cancer[37] consistent with our previous, similar findings in MPM[20-22,37]. The normal physiological role of ARHGDIA is likely to be tissue-specific since ARHGDIA (-/-) knockout mice are viable, but suffer severe and progressive kidney and reproductive system impairment and typically die within a year of birth[38]. While the precise manner in which ARHGDIA regulates apoptosis/cell survival in human cancer is not known, studies in cultured rodent insulinoma cells show that overexpression of ARHGDIA increased cell viability and decreased activated c-Jun N-terminal kinase (JNK) expression following exposure to the apoptosis promoter mycophenolic acid (MPA), whereas knockdown of ARHGDIA (via RNAi) enhanced MPA-induced cell death and increased the activation of JNK[39].

Many types of cancer cells express the M2 isoform of the glycolytic enzyme pyruvate kinase (PKM2), the only gene for which we could not provide evidence linking to either an apoptosis and/or growth arrest phenotype. The PKM2 gene product has been previously linked to cell proliferation *in vivo *[3], presumably by increasing cellular metabolism, and is being evaluated for selective therapeutic targeting based on preferential expression in tumor, and not normal, cells[40]. PKM2 knockdown cell lines in our hands did not display levels of apoptosis or mitosis that differed meaningfully from controls which may not be entirely unexpected given the known role of this enzyme in metabolism and its hypothesized importance in tumor spreading[41], a phenotype we did not measure in the current study. However, as would be expected in a scenario of reduced metabolic activity and decreased proliferation, knockdown of PKM2 was associated with a modest (~15-20%) decrease in cell number (via the Cell Number Index, as a surrogate measure of proliferation) in two of three experiments employing different siRNA molecules, although the results were not statistically significant due primarily to excessive variability.

Although experimentally valid, a clear limitation of our study is in the choice of cell lines modeled, neither of which are mesothelioma-derived, although we have no reason to believe that the basic mechanism of action of any of these genes will differ dramatically among different tumor cell lines, although the strength of effect may. WI38 cells are normal, human, lung-derived fibroblasts. A549 cells are derived from an alveolar (lung) tumor. Several issues governed the choice of these cell lines. MPM tumor cells are not easily amenable to high-throughput assay conditions which we used to rapidly screen a set of four genes to determine which, if any, deserved further analysis in a traditional ("low throughput") one gene/one assay/one cell line experimental design. A549 cells, unlike mesothelioma tumor cell lines, are relatively easy to transfect and are more appropriate for use in a high-throughput assay. Also, there are no established (normal) pleural mesothelial cell lines available to use as a control. The general lack of knowledge surrounding the precise function of the four mesothelioma prognostic genes (ARHGDIA, COBLL1, PKM2, TM4SF1) indicated the importance of examining a normal cell line and WI38, while not ideal, was chosen because it is of similar embryonic origin (i.e., mesoderm) to mesothelial cells lining the thoracic pleura. Finally, despite these limitations, we assert these studies as described are still useful in that they add to the body of knowledge for a set of relatively understudied genes.

Surgical resection for MPM has been reported to improve survival in a subset of patients identified who can be identified pre-operatively using proposed pathological and molecule criteria[20-22]. Our results are consistent with previous data that anti-apoptotic genes are activated in MPM resulting in chemotherapy resistance and tumor cell survival[42,43]. For example, the fact that two of the genes (TM4SF1, COBLL1) are molecular markers for relatively good prognosis patients while the other one gene (ARHGDIA) is a molecular marker for relatively poor prognosis patients suggest that therapies could be tailored and tested in a rational manner to target specific pathways activated in different mesothelioma subsets.

## Conclusion

We have examined the biological function of four validated prognostic genes for MPM and obtained evidence that three of them are involved in some way with protecting the tumor cell from apoptosis. With further validation, these genes may thus provide new potential targets for biological therapy of this deadly disease.

## Competing interests

The authors declare that they have no competing interests.

## Authors' contributions

GJG conceived of the study and produced the experimental data directly and by directing the activities of laboratory technicians. GJG and RB participated in its design and coordination, and all authors analyzed the data, interpreted the results, drafted the conclusions, and read and approved the final manuscript.

## Pre-publication history

The pre-publication history for this paper can be accessed here:

http://www.biomedcentral.com/1471-2407/11/169/prepub
